# Lipocalin-2 participates in sepsis-induced myocardial injury by mediating lipid accumulation and mitochondrial dysfunction

**DOI:** 10.3389/fcvm.2022.1009726

**Published:** 2022-11-07

**Authors:** Weizhuo Liu, Xiaoyu Guo, Lei Jin, Ting Hong, Qianyun Zhang, Fan Su, Yi Shen, Saiqi Li, Bin He

**Affiliations:** ^1^Department of Critical Care Medicine, Shanghai Chest Hospital, Shanghai Jiao Tong University School of Medicine, Shanghai, China; ^2^Center for Cardiopulmonary Translational Medicine, Shanghai Chest Hospital, Shanghai Jiao Tong University School of Medicine, Shanghai, China

**Keywords:** lipocalin-2, sepsis, myocardial injury, lipid metabolism, mitochondrial dysfunction

## Abstract

**Background:**

Sepsis-induced cardiomyopathy (*SIC*) is one major cause of death for sepsis but lacks timely diagnosis and specific treatment due to unclear mechanisms. Lipocalin-2 (LCN-2) is a key regulator of lipid metabolism which has been recently proved closely related to sepsis, however, the relationship between LCN-2 and septic myocardial injury remains unknown. We aim to explore the role of LCN-2 in the pathological progress of *SIC* based on clinical and laboratory evidence.

**Methods:**

Consecutive patients admitted to the intensive care unit (ICU) from August 2021 to April 2022 fulfilling the criteria of severe sepsis were included. The level of LCN-2 in plasma was assayed and analyzed with clinical characteristics. Biostatistical analysis was performed for further identification and pathway enrichment. Mouse model for *SIC* was thereafter established, in which plasma and tissue LCN-2 levels were tested. RNA sequencing was used for verification and to reveal the possible mechanism. Mitochondrial function and intracellular lipid levels were assayed to further assess the biological effects of targeting LCN-2 in cardiomyocytes with small interference RNAs (siRNAs).

**Results:**

The level of LCN-2 in plasma was markedly higher in patients with severe sepsis and was associated with higher cardiac biomarkers and lower LVEF. In the *in vivo* experiment, circulating LCN-2 from plasma was found to increase in *SIC* mice. A higher level of LCN-2 transcription in myocardial tissue was also found in *SIC* and showed a clear time relationship. RNA sequencing analysis showed the level of LCN-2 was associated with several gene-sets relevant to mitochondrial function and lipid metabolism-associated pathways. The suppression of LCN-2 protected mitochondrial morphology and limited the production of ROS, as well as restored the mitochondrial membrane potential damaged by LPS. Neutral lipid staining showed prominent lipid accumulation in LPS group, which was alleviated by the treatment of siLCN2.

**Conclusion:**

The level of LCN-2 is significantly increased in *SIC* at both circulating and tissue levels, which is correlated with the severity of myocardial injury indicators, and may work as an early and great predictor of *SIC*. LCN-2 probably participates in the process of septic myocardial injury through mediating lipid accumulation and affecting mitochondrial function.

## Graphical Abstract

Study flowchart and schematic diagram for the role of LCN-2 in sepsis-induced myocardial injury 
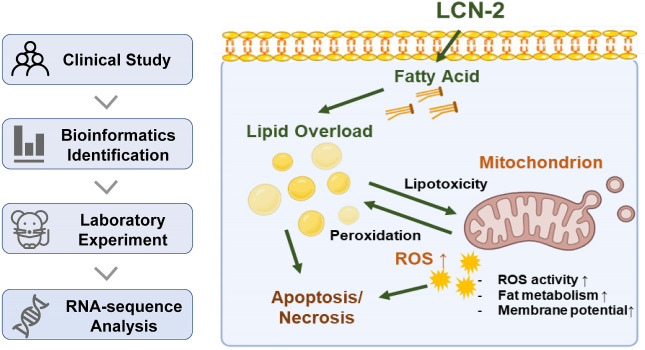

## Introduction

Sepsis is the life-threatening organ dysfunction caused by a dysregulated host response to infection (1, 2). Cardiac dysfunction or injury caused by sepsis, i.e., sepsis-induced cardiomyopathy (*SIC*), manifesting clinically as decreased myocardial contractility, weakened response to cardiac preload, etc., is one of the leading causes of death for sepsis (3, 4). However, timely diagnosis and potential therapeutic targets still remain a major challenge in the management of *SIC*, in which discovering novel biomarkers can be helpful (4).

Lipid metabolism is vital for maintaining the normal function of mitochondria which plays an important role in *SIC* (5, 6). The disorder of lipid metabolism increases reactive oxygen species (ROS) production and fission of mitochondrial, leading to mitochondrial dysfunction and cell apoptosis (7–9). Recent studies have revealed the disorder of lipid metabolism in several kinds of sepsis-induced organ injury (10, 11), which has also been observed in septic myocardial tissue in our previous work (12), however, *via* unclear mechanisms.

Lipocalin-2 (LCN-2), also known as neutrophil gelatinase-associated lipocalin (NGAL), is an innate immune protein mainly responsible for the transportation of lipids, iron, etc., acting as an upstream regulator of lipid metabolism (13). It has broad expression in various tissues and cell types including cardiomyocytes and endothelial cells, which has been recognized as an emerging player in different physiological and pathological processes, including iron homeostasis, mitochondria function, inflammation, microbial infection, etc. (14, 15).

It has been found that LCN-2 participates in the development of heart failure, myocardial hypertrophy, myocardial ischemia-reperfusion injury, chemotherapy-related cardiac function injury, and other heart diseases (16–18). Recent studies have also shown its close correlation to the severity of sepsis (19, 20), the relationship between LCN-2 and septic myocardial injury, however, remains unclear. In this study, we aim to explore the role of LCN-2 in the pathological progress of septic myocardial injury based on clinical and laboratory evidence, as well as its potential mechanism.

## Materials and methods

### Materials and reagents

Materials and reagents were included in Supplementary material.

### Clinical study population and data collection

Patients admitted to the intensive care unit (ICU) from August 2021 to April 2022 who fulfilled the criteria for severe sepsis or septic shock (1) and were 18 years or older were consecutively included. Patients with previous myocardial infarction, heart failure with reduced ejection fraction, moderate to severe valve diseases and phase 4/5 chronic kidney disease, or those with a history of percutaneous coronary angioplasty or coronary bypass surgery, or those without risk evaluation or proper resuscitation therapy in accordance with the guide for sepsis bundle, were excluded from the study. Those with missing data pertaining to sex, date of birth, diagnosis or lack of important examination data were also ruled out. A control group was set up with individuals without any evidence of sepsis and free of any heart diseases at baseline. The study was approved by the Institutional Review Board (IRB) for Human Research at Shanghai Chest Hospital Shanghai Jiao Tong University in August 2021 entitled Early Diagnosis and Prognostic Assessment in Sepsis-Induced Myocardial Injury (approval No. KS(Y)22061), and conducted in accordance with the ethical standards of the IRB on human research and with the Helsinki Declaration of 1975. Informed consent was obtained from patients at the time of enrollment.

The clinical history of all patients including age, gender, comorbidities, and laboratory results were retrieved from the hospital information system. In both groups, plasma was centrifuged within 30 min and plasma was stored at –80°C for subsequent analysis. Plasma LCN-2 was determined using Human Lipocalin ELISA Kit (RayBiotech, United States). Plasma chemistry, myocardial enzyme and markers, arterial blood gas analyses, and other laboratory tests including C-reactive protein (CRP), procalcitonin (PCT), etc. were assayed at the same time by routine laboratory methods. All subjects had continuous electrocardiogram (ECG) monitoring and arterial cannulation for invasive pressure monitoring as part of standard clinical practice. The results of echocardiographic tests performed within 3 days were also acquired.

### Bioinformatic identification based on gene expression omnibus database

Biostatistical analysis of transcriptome array datasets was used to further identify critical genes and enrich related signaling pathways. Two microarray profile datasets GSE28750 and GSE57065 of raw gene expression downloaded from gene expression omnibus (GEO) datasets and processed *via* GEO2R^1^ (21), were investigated to screen key genes in sepsis. Differential expression genes (DEGs) were identified by calculating fold changes and *P*-values between sepsis and control groups. In this study, the cut-off criteria are *P*-value < 0.01 and | log fold change (FC)| > 2. Subsequently, the overlapping DEGs consisting of common up-regulated and common down up regulated were presented through Venn diagrams.

Gene Ontology (GO) (22) analysis and Kyoto Encyclopedia of Gene and Genomes (KEGG) (23) pathway enrichment analyses were applied to study DEGs at the function level. GO annotations include three items: molecular functions (MF), biological processes (BP), and cellular components (CC), and KEGG is a set of genes and protein pathways with close interaction relationships. In this study, an online visualized tool^2^ was used for GO and pathway enrichment. STRING database was applied to build a protein-protein interaction (PPI) network for discovering hub genes. *P* < 0.05 was set as the cut-off criterion.

### Model establishment

The cellular model of *SIC* was established by lipopolysaccharide (LPS, Sigma-Aldrich, United States) incubation based on reported protocol (12, 24). Briefly, H9C2 cells were cultured in DMEM containing 10 μg/ml LPS for 12 h. Morphology of cardiomyocytes was observed. C57BL/6 mice weighing 25–28 g (GemPharmatech Co., China) were raised in a specific pathogen-free (SPF) environment. LPS was injected intraperitoneally (i.p.) at a dosage of 20 mg/kg for 12 h to induce cardiac injury. Cardiac markers were tested to validate the establishment of models. All experimental procedures and animal studies were approved by the Animal Ethics Committee of Shanghai Chest Hospital.

### Assay of plasma lipocalin-2 level in mice model

Plasma was centrifuged for 30 min and plasma was stored at –80°C for subsequent analysis. Plasma LCN-2 was determined at 12 h from the onset of *SIC* using Mouse Lipocalin ELISA Kit (RayBiotech, United States) by ELISA test. Mean values were calculated and used for statistical analyses.

### Quantitative polymerase chain reaction of myocardial tissue

The gene expression of myocardial tissue was determined by RT-quantitative polymerase chain reaction (qPCR) with SYBR Green. Relative expression was quantified to Rplp0 as the internal standard control. All primer sequences are listed in Supplementary Table 1.

### RNA sequencing and analysis

RNA was extracted from heart tissues of *SIC* mice at 12 h after LPS stimulation that was grown under the appropriate conditions using the RNeasy mini kit (Qiagen, United States). After that, RNA sequencing (RNA-seq) was performed by GENEWIZ (Suzhou, China). Genes with a | fold-change (FC)| > 2 and *P* < 0.05 were defined as significant differential expressed genes for further analysis. Gene Set Enrichment Analysis (GSEA) is a useful tool for the correlation analysis of different gene sets. We also used GSEA for function enrichment grouped by the high or low level (using the cut-off value calculated by Receiver Operator Characteristic curve) of LCN-2 in the RNA-sequence analysis.

### Staining of cardiomyocyte

The intracellular lipid of H9C2 cells was detected and analyzed at 12 h after LPS induction using HCS LipidTOX™ (Invitrogen, United States). Laser scanning confocal microscope technology was then performed. The mitochondrial morphology and ROS level were assessed by MitoTracker™ Red CMXRos (Invitrogen, United States) and DCFH-DA assessment (Invitrogen, United States) at 12 h after LPS stimulation, as well as the estimation of the mitochondrial membrane potential using the JC-1 probe (Invitrogen, United States).

### Statistical analysis

Clinical variables were expressed as a percentage (%) for categorical variables, mean with standard deviation (SD) for normally distributed continuous variables, and median with interquartile rate (IQR) for discontinuous variables. To compare categorical variables, chi-square test or Fisher’s exact test was used; to compare continuous variables, an unpaired two-tailed *t*-test or Mann–Whitney *U*-test was used. Patients with severe sepsis were then divided into low/high LCN-2 subgroups according to the 75th percentile following classic classification method (25, 26) in the following subgroup analysis.

In laboratory verification, data was presented as the mean ± SEM at least three duplications of different samples. Student’s *t*-test was applied for analysis between two groups and one-way analysis of variance (ANOVA) was used for comparisons between multiple groups. Statistical analysis was performed with Stata 16.0 software. GraphPad Prism 7.0 (GraphPad Software Inc., United States) were used to analyze and illustrate the data. Differences with *p*-values < 0.05 were considered statistically significant.

## Results

### Clinical data analysis

**FIGURE 1:**
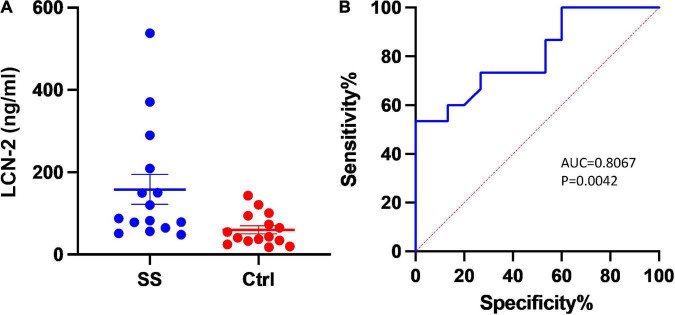
Plasma level of LCN-2 between groups. **(A)** Scatter plots of plasma LCN-2 levels. **(B)** The receiver operating characteristic analysis (ROC) of LCN-2 to predict severe sepsis and sepsis shock. AUC, Area under curve; Ctrl, Controls; SS, Severe sepsis.

Fifteen patients with severe sepsis or septic shock meeting entry criteria were enrolled into the trial. A control group of fifteen individuals was established at a 1:1 ratio. Baseline characteristics of enrolled patients were listed in Table 1. In addition to the common indicators (white cell count, C-reactive protein, etc.) for indications that differed significantly between the two groups, the level of LCN-2 in plasma was also markedly higher in SS (median: 87.8 [64.5, 209.2] ng/ml, mean: 158.32 ± 140.61 ng/ml) than in control group (median: 43.2 [32.6, 94.3] ng/ml, mean: 60.19 ± 38.67 ng/ml) with statistical difference (*P* = 0.015). Scatter plots of plasma LCN-2 levels showed it fluctuated greatly in patients with severe sepsis (Figure 1A), requiring further subgroup analysis. The ROC revealed LCN-2 was a predictor for sepsis (cut-off value = 75.57 ng/ml, AUC = 0.8067, *P* = 0.0042) (Figure 1B).

**TABLE 1:** Baseline characteristics of the patients.

Variable	Severe sepsis/Sepsis shock	Control group	*P-value*
Age (years)	70.6 ± 7.5	65.8 ± 5.8	0.06
Male gender (n,%)	14 (93.3)	13 (86.7)	0.54
Hypertension (n,%)	6 (40.0)	4 (26.7)	0.44
Diabetes (n,%)	3 (20.0)	2 (13.3)	0.62
WBC (*10^9^)	20.00 ± 5.97	11.09 ± 2.96	<0.001
Neutrophil % (%)	92.78 ± 0.03	84.11 ± 0.07	<0.001
C-reactive protein (mg/l)	236.64 ± 66.49	89.96 ± 74.66	<0.001
Procalcitonin (μg/L)	2.73 [2.12, 4.61]	0.19 [0.12, 0.31]	<0.001
LCN-2 (ng/ml)	87.81 [64.55, 209.16]	43.16 [32.56, 94.36]	0.015*
Pro-BNP (pg/ml)	3060 [2180, 4800]	368 [295, 527]	0.025
Serum creatine (μmol/l)	89.1 [67.9, 285.0]	65.3 [58.0, 78.0]	0.019
Bilirubin (μmol/L)	35.3 [16.3, 76.3]	19.1 [11.9, 25.08]	0.040
Mechanical ventilation (%)	12 (80.0)	4 (26.7)	0.003
Renal replacement therapy (%)	4 (26.7)	1 (6.7)	0.032

Clinical variables were expressed as a percentage (%) for categorical variables, mean with SD for normally distributed continuous variables, and median with IQR for discontinuous variables. LCN-2, Lipocalin-2; Pro-BNP, pro-type-B natriuretic peptide. *P < 0.05.

In the subgroup analysis of sepsis group, the sample was categorized according to the 75th percentile of LCN-2 (179 μg/l) as mentioned above into Low LCN-2 group (LCN-2 < 179 μg/l) and High LCN-2 group (LCN-2 ≥ 179 μg/l). Vital signs indicators, along with important cardiac-related biomarkers were compared and analyzed between two subgroups in Table 2. The High LCN-2 group had a higher APACHE II score (*P* = 0.006) on admission. All patients required inotropic or vasopressor support in both subgroups. Patients with high level of LCN-2 have a higher level of pro-B-type natriuretic peptide (pro-BNP) (6490 vs. 2510 pg/ml, *P* = 0.005), cardiac troponin I (cTNI) (0.135 vs. 0.02 ng/l, *P* = 0.067), myoglobin (MYO) (487.7 ± 156.9 ng/l, *P* = 0.055) and CK-MB (12.60 vs. 2.05 ng/l, *P* = 0.009) than those with low level of LCN-2.

**TABLE 2:** Cardiac-related characteristics in Low/High LCN-2 subgroup.

Variable	Low LCN-2 (<179)	High LCN-2(≥ 179)	*P-value*
APACHE-II score	25.1 ± 5.7	36.0 ± 5.9	0.006
Length of stay in ICU	17.0 ± 8.0	30.8 ± 17.0	0.048
Death (%)	3 (27.3)	2 (50)	0.409
Inotropic/vasopressor support (%)	11 (100)	4 (100)	>0.999
CVP (mmHg)	4.1 ± 2.2	9.7 ± 3.9	0.003
MAP (mmHg)	57.9 ± 10.3	43.8 ± 9.5	0.033
Pro-BNP (pg/ml)	2510 [1420, 3390]	6490 [4290, 9790]	0.005
cTNI (ng/l)	0.02 [0.01, 0.04]	0.135 [0.09, 1.51]	0.067
MYO (ng/l)	156.9 [33.2, 339.4]	487.7 [212.7, 2219.4]	0.055
CK-MB (ng/l)	2.05 [0.80, 5.09]	12.60 [10.05, 24.11]	0.009
Arrythmia (%)	2 (18.2)	3 (75.0)	0.039
Ejection fraction (%)	64 [62, 65]	56 [52, 59]	0.019

CK-MB, creatine kinase MB isoenzyme; cTNI, cardiac troponin I; CVP, central venous pressure; MAP, mean venous pressure; MYO, myoglobin.

A lower ejection fraction (EF) was more common in the High LCN-2 group than in the Low LCN-2 group (*P* = 0.019). Arrhythmia was more frequently observed in High LCN-2 group (75.0%) than in Low LCN-2 group (18.2%) (*P* = 0.039), among which new-onset atrial fibrillation was most common.

### Differentially expressed genes identification and analysis based on gene expression omnibus database

**FIGURE 2:**
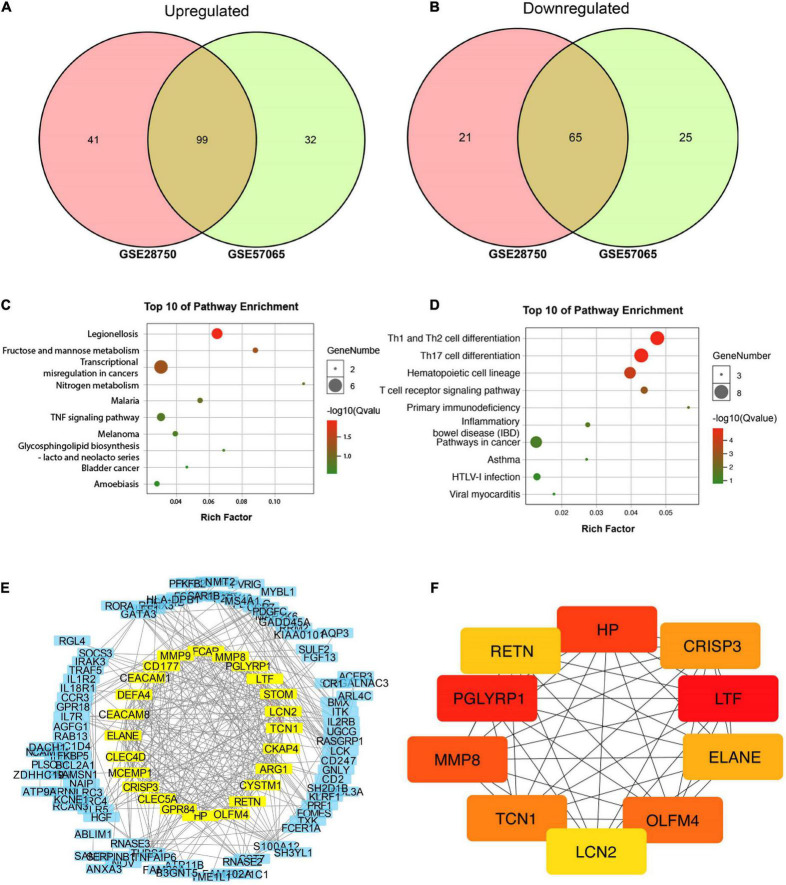
Bioinformatic identification based on gene expression omnibus (GEO) database. **(A,B)** Venn diagram of 99 common upregulated and 65 common downregulated DEGs. **(C)** Top 10 pathways enriched by GO. **(D)** Top 10 pathways enriched by KEGG. **(E)** Protein-protein interaction (PPI) network for discovering hub genes. **(F)** Top 10 molecules in network string interactions ranked by maximal clique centrality (MCC) method.

GSE28750 and GSE57065 were used for screening differentially expressed genes (DEGs) for bioinformatic analysis. GSE28750 was consisted of 38 sepsis samples and 20 control samples, and GSE57065 covered 28 sepsis samples and control samples. The DEGs were initially prescreened according to the strict criteria of *P*-value < 0.01 and | log FC| > 2. Finally, 99 upregulated and 65 downregulated common DEGs were recognized for further analysis (Figures 2A,B). In addition, the common DEGs were presented in Supplementary Table 2.

All 164 DEGs consisted of 99 upregulated DEGs and 65 downregulated DEGs were analyzed *via* online tools, the results of which were presented in Figure 2. As is shown in Supplementary Tables 3, 4, for biological processes (BP), upregulated DEGs were enriched in myeloid leukocyte activation, neutrophil degranulation, neutrophil activation involved in immune response, neutrophil-mediated immunity and neutrophil activation. For cellular components (CC), upregulated DEGs were enriched in secretory granule, specific granule, secretory vesicle, tertiary granule and cytoplasmic vesicle part. For molecular functions, upregulated DEGs were particularly gathered in protein homodimerization activity, carbohydrate kinase activity, 6-phosphofructo-2-kinase activity, catalytic activity and fructose-2,6-bisphosphate 2-phosphatase activity (Supplementary Figure 1).

Moreover, DEGs related pathways were enriched by GO (Figure 2C) and KEEG (Figure 2D) pathway enrichment. Then STRING database was applied to build a PPI network for discovering hub genes (Figure 2E). Ten hub genes were obtained in network string interactions ranked by maximal clique centrality (MCC) method (Figure 2F), among which LCN-2 showed preferable significance, along with several molecules reported to be related to lipid or other metabolism including PGRPs, OLFM4, RETN etc.

### Validation on animal models

**FIGURE 3:**
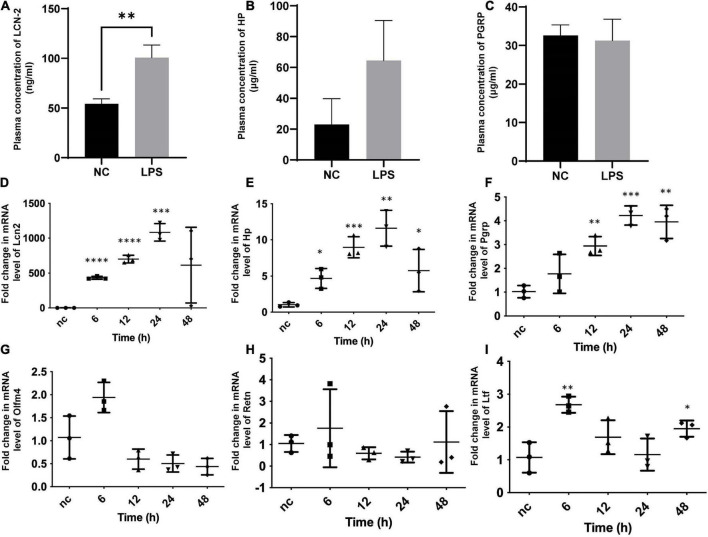
Plasma assay for circulating concentration and qPCR of myocardial tissue for cardiac mRNA determination in *SIC* mouse. **(A–C)** Plasma concentration of LCN-2, HP, PGRP of both groups at 12 h after the induction of LPS. **(D–I)** Fold change in mRNA level of Lcn2, Hp, Pgrp, Olfm4, Retn, Ltf of myocardial tissue at different times testing by qPCR. Hp, haptoglobin; Lcn2, lipocalin-2; Ltf, lactotransferrin; Olfm4, olfactomedin-4; Pgrp, peptidoglycan recognition protein-1; Retn, resistin. *P < 0.05, **P < 0.01, ***P < 0.001, and ****P < 0.0001.

The mouse model of *SIC* group (*n* = 6) and control groups (*n* = 6) were successfully established. Together with LCN-2, three secreted molecules chosen from the results of the bioinformatic analysis were assayed from the plasma at 12 h after LPS stimulation (Figures 3A–C). The level of LCN-2 was significantly higher (100.7 ± 31.0 ng/ml) in *SIC* group than in control group (54.2 ± 12.6 ng/ml) (*P* = 0.007).

For further identification and exploration, the mRNA of myocardial tissue was extracted at different times (0, 6, 12, 24, 48 h) from the onset of *SIC* (Figures 3D–I), LCN-2 and other molecules chosen from the results of the bioinformatic analysis were assayed for each stage. LCN-2 demonstrated a high level of transcription in the heart tissue and showed a clear time relationship with significant change at the 6th hour and peak at the 24th hour.

### RNA sequencing and analysis

**FIGURE 4:**
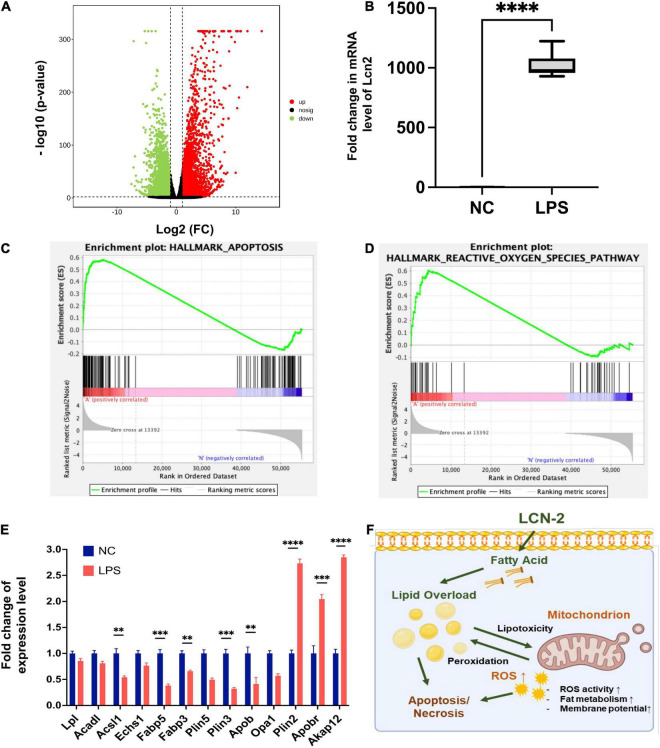
**(A)** Volcano plot of DGEs of mouse myocardial tissue between groups by RNA sequence at 12 h after LPS stimulation. **(B)** Fold change level of LCN-2 in myocardial tissue. **(C,D)** GSEA analysis for function enrichment grouped by the high or low level of LCN-2 in the RNA-sequence analysis. **(E)** Molecules related to fatty acid uptake and β-oxidation changing obviously in LPS group. **(F)** Possible mechanism by which LCN-2 participates in septic myocardial injury *via* lipid metabolism disorder and mitochondrial dysfunction. **P < 0.01, ***P < 0.001, and ****P < 0.0001.

RNA sequencing analysis of myocardial tissue from 12 mice (LPS, *n* = 6; NC, *n* = 6) was shown in Figures 4A,B and Supplementary Figure 2, DEGs consisted of 2127 upregulated DEGs and 1687 downregulated DEGs were detected (Figure 4A). Pathways enrichment by GO and KEGG were shown in Supplementary Figure 2. Fold change level of LCN-2 in myocardial tissue in LPS group was almost 1,000 times than NC group (*P* < 0.001) (Figure 4B). GSEA analysis for function enrichment grouped by the high or low level of LCN-2 (using the cut-off value calculated by Receiver Operator Characteristic curve, FPKM: 913.8) showed the level of LCN-2 was strongly associated with cell apoptosis, ROS, adipogenesis and cholesterol homeostasis (top 20 pathway enriched) (Figures 4C,D and Supplementary Figures 3A,B). In addition, many fatty acid metabolism-related molecules, including LPL, ACSL1, FABPs, PLINs, etc., which were relevant with mitochondrial function and previously reported to be mediated by LCN-2, changed obviously in LPS group (Figure 4E and Supplementary Figure 3C). The level of STAT3 also significantly increased, which was reported to be the transcription factor of LCN-2 (Supplementary Figure 3D). Thus, we speculated LCN-2 could probably participate in *SIC* and be associated with myocardial injury.

### Functional verification for mitochondria damage and lipid accumulation

**FIGURE 5:**
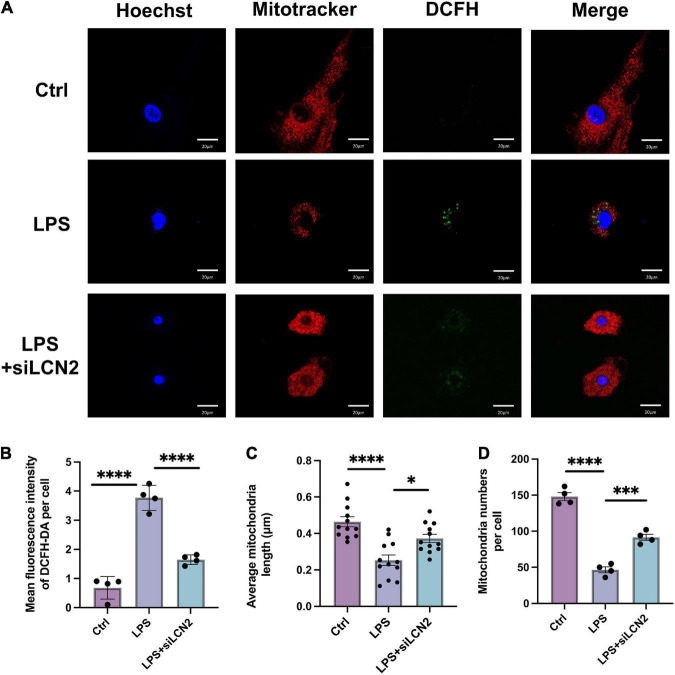
The suppression of LCN-2 protected mitochondrial morphology and limited the production of ROS. **(A)** Mitochondrial morphology (MitoTracker, red) and ROS level (DCFH-DA, green) of LPS damaged H9C2 cardiomyocytes pretreated with siLCN2 (*n* = 3; scale bar = 20 μm). **(B)** Mean fluorescence intensity of DCFH-DA (*n* = 4). **(C)** Average mitochondria length of cardiomyocytes in TEM (*n* = 12). **(D)** Average number of mitochondria of cardiomyocytes (*n* = 4). Data are presented as mean ± SEM. Statistical analysis was *via* one-way ANOVA with Bonferroni multiple-comparison correction; *P < 0.05, ^**^P < 0.01, ^***^P < 0.001, and ^****^P < 0.0001.

The mitochondrial morphology and ROS level were assayed by MitoTracker™ and DCFH-DA assessment at 12 h after LPS stimulation, which showed disordered mitochondria with abnormal appearance and increased ROS level after LPS induction. By contrast, when cardiomyocytes were pretreated with LCN2-siRNA (siLCN2), the normal mitochondrial structure was restored to some extent and ROS level was decreased (Figure 5).

**FIGURE 6:**
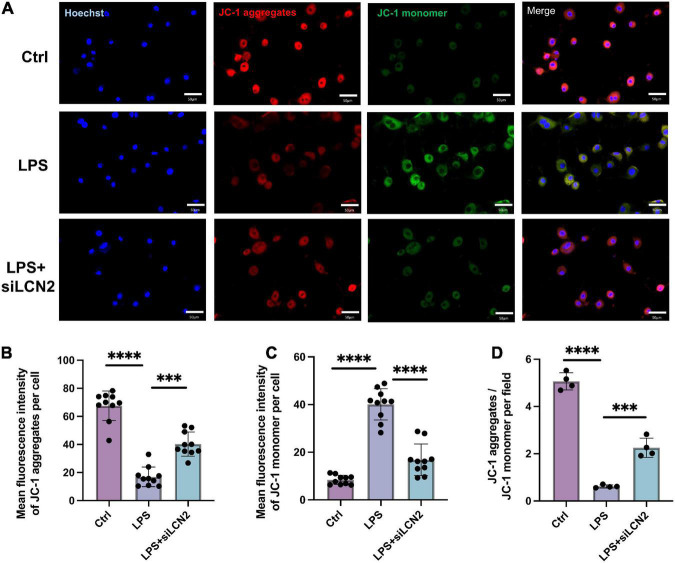
Silence of LCN-2 restored the mitochondrial membrane potential damaged by LPS. **(A)** Mitochondrial membrane potential (JC-1) of LPS damaged H9C2 cardiomyocytes pretreated with siLCN2. Red indicated JC-1 aggregates and green indicated JC-1 monomer (*n* = 3, scale bar = 50 μm). **(B–D)** Quantitative results of mitochondrial membrane potential. Data are presented as mean ± SEM. Statistical analysis was *via* one-way ANOVA with Bonferroni multiple-comparison correction; ^**^P < 0.01, ^***^P < 0.001, and ^****^P < 0.0001.

Estimation of the mitochondrial membrane potential using the JC-1 probe demonstrated that the LPS stimulation obviously decreased the mitochondrial membrane potential (decreased JC-1 aggregates, red; increased JC-1 monomer, green), suggesting mitochondrial function damage. In contrast, treatment by siLCN2 significantly increased the level of mitochondrial membrane potential (JC-1 aggregates, red), indicating restored mitochondrial function after siLCN2 treatment (Figure 6).

**FIGURE 7:**
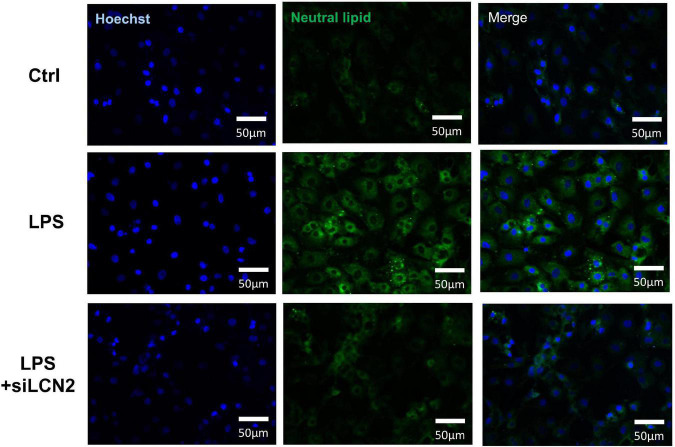
Neutral lipid staining of LPS damaged H9C2 cardiomyocytes at 12h after LPS stimulation. The inhibition of LCN-2 alleviated the lipid accumulation in H9C2 cells caused by LPS stimulation.

Lipid metabolism is indispensable for maintaining normal mitochondria function. Neutral lipid staining was then performed at 12 h after the stimulation of LPS using HCS LipidTOX™ and showed prominent lipid accumulation in LPS group, which was alleviated by the treatment of siLCN2 (Figure 7 and Supplementary Figure 4 for quantification), indicating LCN-2 may participate in the process of septic myocardial injury *via* inducing lipid accumulation and mitochondrial dysfunction (Figure 4F). Further explorations for detailed mechanisms could be conducted in the following studies.

## Discussion

Sepsis-induced cardiomyopathy is one of the leading causes of death for sepsis (3). However, it often lacks timely diagnosis and specific treatment due to the uncertain mechanism and diagnostic criteria (4). Thus, it is helpful to find valuable biomarkers assisting the diagnosis and treatment of *SIC*.

LCN-2 has been recognized as a promising inflammatory marker involved in many acute inflammatory damages in many diseases such as acute kidney injury, ischemic stroke, myocardial ischemia-reperfusion injury, etc. (16, 18, 27, 28). It participates in various pathological processes *via* multiple immune or inflammation-related mechanisms in diverse situations, leading to the inconsistent results based on different cell types and conditions (29–31). In this study, we focus on the acute-phase of sepsis-induced myocardial injury where the role of LCN-2 remains unclear, and found a strong relationship with myocardial damage based on clinical and laboratory evidence.

In current study, the level of LCN-2 was much higher in plasma in severe sepsis patients which is similar to previous reports (19, 32). In the subgroup analysis, amongst the population with sepsis, patients with a higher level of LCN-2 demonstrated higher cardiac markers and lower LVEF, etc., indicating LCN-2 was potentially associated with septic myocardial injury.

Microarray analysis in transcriptome profile has been used as a valuable laboratory tool to identify critical genes and signaling pathways involved in pathogenesis and solve the limitation of clinical sample size (33). Transcriptome profiling usually yields a considerable number of potential differential transcripts, which is a challenge to identify the critical genes. A pooled biostatistical analysis of a transcriptome array dataset which combines multiple species may help screen conservative and important genes involved in a given disorder (34). In the bioinformatic analysis, LCN-2 showed great significance, along with several modules reported to be related to lipid or other metabolism.

*In vivo*, circulating LCN-2 from plasma was verified to increase in *SIC* mice. In order to seek a more direct connection with myocardial tissue, the level of LCN-2 from septic myocardial tissue was tested, which as a result, demonstrated a high level of transcription in the heart tissue, and showed a clear time dependence with significant change at the 6th hour and peak at 24th hour, providing more direct evidence of the relationship between LCN-2 and LPS-induced myocardial injury.

Mitochondrial dysfunction is a well-recognized mechanism in *SIC* (3, 4). In the RNA-sequence analysis, the level of LCN-2 was strongly associated with cell apoptosis, ROS, using GSEA for function enrichment grouped by the high or low level of Lcn2. Moreover, the suppression of LCN-2 protected mitochondrial morphology and limited the production of ROS, as well as restored the mitochondrial membrane potential damaged by LPS, indicating LCN-2 may participate the process of mitochondrial dysfunction.

Lipid metabolism is indispensable for maintaining normal mitochondria function (5, 35), while lipotoxicity, often caused by lipid overload, has been reported in multiple cardiac pathological processes by influencing mitochondrial function (36–38). LCN-2 is reported to be responsible for the transportation of small lipophilic molecules such as lipids (13, 14). Through analyzing the change of differential genes in LPS-induced cardiac injury by RNA sequencing, LCN-2 and other relevant molecules related to fatty acid uptake and β-oxidation changed obviously which participated in mitochondrial activity. In addition, neutral lipid staining showed prominent lipid accumulation in LPS group, which was alleviated by the suppression of LCN-2, indicating LCN-2 may participate in the process of septic myocardial injury *via* inducing lipid accumulation and mitochondrial dysfunction. Further explorations for detailed mechanisms could be conducted in the following studies.

## Conclusion

The level of LCN-2 is significantly increased in *SIC* at both circulating and tissue level, which is correlated with the severity of myocardial injury indicators, and may work as an early and great predictor of *SIC* in clinical practice. LCN-2 probably participates in the process of septic myocardial injury through mediating lipid accumulation and affecting mitochondrial function.

## Data availability statement

The datasets presented in this study can be found in online repositories. The names of the repository/repositories and accession number(s) can be found in the article/Supplementary material.

## Ethics statement

The studies involving human participants were reviewed and approved by Institutional Review Board (IRB) for Human Research at Shanghai Chest Hospital, Shanghai Jiao Tong University. The patients/participants provided their written informed consent to participate in this study. The animal study was reviewed and approved by Animal Ethics Committee of Shanghai Chest Hospital.

## Author contributions

WL, XG, SL, and BH designed the study. LJ was responsible for the collection of clinical samples. BH supervised the whole work. WL and XG drafted the manuscript. All authors contributed to the data collection or analysis, reviewed it for important scientific content, and approved the final version of this manuscript.

## Footnotes

1
http://www.ncbi.nlm.nih.gov/geo/geo2r

2
https://www.omicshare.com/tools/
