# Supplementary material for: Lipocalin-2 participates in sepsis-induced myocardial injury by mediating lipid accumulation and mitochondrial dysfunction
Source: Front Cardiovasc Med. 2022 Nov 7;9:1009726. doi: 10.3389/fcvm.2022.1009726 (PMC9676239; doi:10.3389/fcvm.2022.1009726)
Supplement: Supplementary file 1 [file Data_Sheet_1.docx]

Supplementary Material

**Lipocalin-2 Participates in Sepsis-Induced Myocardial Injury by Mediating Lipid Accumulation Disorder and Mitochondrial Dysfunction**

**Key Words:** Lipocalin-2, Sepsis, Myocardial Injury, Lipid Metabolism, Mitochondrial Dysfunction.

The PDF file includes:

1. Materials and Reagents
2. Supplementary Table S1-4
3. Supplementary Figure S1-4
4. **Materials and Reagents**

| Reagents | Company | Catalog No. |
| --- | --- | --- |
| Human Lipocalin ELISA Kit | RayBiotech | United States |
| Mouse Lipocalin ELISA Kit | RayBiotech | United States |
| MitoTracker™ Red CMXRos | Invitrogen | M7512 |
| Hoechst 34850 | Invitrogen | H21486 |
| DCFH-DA | Invitrogen | S0033M |
| JC-1 probe | Invitrogen | M34152 |
| Fluorescein isothiocyanate (FITC) | Invitrogen | F1907 |
| HCS LipidTOX™ | Invitrogen | MP34475 |
| Fetal bovine serum | Gibco | 10100147 |
| HyClone Dulbecco's Modified Eagle Medium (DMEM) with low glucose | Cytiva | SH30021.01 |
| 0.25% Trypsin-EDTA | Gibco | 25200072 |
| 0.25% Trypsin | Gibco | 15050057 |
| PBS without calcium, magnesium | Cytiva | SH30256.01 |
| HBSS without calcium and magnesium | Cytiva | SH30588.0 |
| EDTA | Merck | E6758 |
| Complete Protease Inhibitor Cocktail | Merck | 04693116001 |
| PhosSTOP™ | Merck | 04906845001 |
| Complete Lysis-M buffer | Roche | 04719956001 |

1. **Supplementary Table S1-S4**

**Supplementary Table S1**：

Primer sequences for qPCR of myocardial tissue.

| Official Symbol | Gene ID | Upper Primer Sequence | Lower Primer Sequence |
| --- | --- | --- | --- |
| LCN-2 | 16819 | 5' CTC AAG GAC GAC  AAC ATC 3' | 5' CGC CTG AAC CAT TGG  GTC TCT G 3' |
| HP | 15439 | 5' CGA CCA GGA CAA  GTG TGT G 3' | 5' CAA CGG GAC TCG TCA  AGT T 3' |
| PGRPs | 21946 | 5' TGG AGA GGA CGG  TCA TGT CTA 3' | 5' CCT GTG TGG TCA CCC  TTG AT 3' |
| OLFM4 | 380924 | 5' ACC CAC CAC ATA  GAA CCA TTG 3' | 5' ATT TGC TCT CAT GTC  CAC CTC 3' |
| RETN | 57264 | 5' CAA ACA AGA CTT  CAA CTC CCT 3' | 5' AAG ACT GCT GTG CCT  TCT G 3' |
| LTF | 17002 | 5' AGT TTT GCC TGT TCC  AGT CTA A 3' | 5' TTT CCC AGA TAC TTC  TCC GAT 3' |

**Supplementary Table S2**：

DEGs identification and analysis based on GSE28750 and GSE57065 database

| DEGs | Gene symbols |
| --- | --- |
| Upregulated(99) | FKBP5, SERPINB1, PFKFB3, LCN2, B3GNT5, ASPH, UGCG, RETN, SORT1, CEACAM1, TNFAIP6, S100A12, GYG1, TDRD9, SAMSN1, OPLAH, GRB10, AGFG1, IDI1, PLSCR1, HPGD, RRM2, ATP11B, STOM, FGF13, CLEC4D,  VNN 1.00, NLRC4, KIAA0101, IL18R1, KCNE1, RNASE2, CA1, THBS1, FAM20A, PCOLCE2, SOCS3, METTL7B, ANKRD22, SULT1B1, NAIP, PGLYRP1, CLEC5A, MMP8, LTF, IL10RB-AS1, METTL9, CD177, PFKFB2, TCN1, CA4, UPP1, ANXA3, ATP9A, LILRA5, ELANE, GADD45A, ADAM9, CRISP3, DEFA4, HGF, RGL4, ST6GALNAC3, DACH1, OLFM4, YOD1, ARG1, FCGR1B, SAR1B, RNASE3, MS4A4A, CST7, PDGFC, CNIH4, IRAK3, OLAH, CYP1B1, MCEMP1, TLR5, ZDHHC19, ST3GAL4-AS1, CEACAM8, BCL2A1, FCAR, CR1, RAB13, HK3, ACER3, GPR84, NSUN7, ATP6V1C1, IL1R2, SMPDL3A, BMX, CKAP4, CYSTM1  MMP9, MAP2K6, HP |
| Downregulated(65) | PRF1, TRBC1, IL7R, PVRIG, FAM102A, ARL4C, RUNX3, IFIT1, PRKCH, IL2RB, CD3E, MYBL1, YME1L1, LEF1, SCML4, CD247, ITK, TXK, PASK, TBC1D4, EVL, HLA-DPB1, IGHM, RCAN3, RASGRP1, CAMK4, TRAC, TRAF5, CCR3, NMT2, P2RY10, NOV, SH3YL1, LCK, CD2, TMEM204, ATP8B2, MS4A1, ABLIM1, CCR7, HLA-DPA1, EOMES, TGFBR3, SH2D1B, NLRC3, BCL11B, GATA3, RORA, GPR18, FCMR, FCER1A, SULF2, DYRK2, LBH, LRRN3, NELL2, AQP3, SGK223, TRDV3, FLT3LG, GNLY, SAMD3, RPS6KA5, KLF12, KLRF1 |

**Supplementary Table S3**：

Results of the GO enrichment analysis of DEGs (Top 5)

| Oncology | GO ID | GO Term | Count | P-value | FDR |
| --- | --- | --- | --- | --- | --- |
| Upregulated |  | | | | |
| BP | GO:0002274 | myeloid leukocyte activation | 38 | 9.63E-29 | 2.78E-25 |
|  | GO:0043312 | neutrophil degranulation | 34 | 3.64E-28 | 4.94E-25 |
|  | GO:0002283 | neutrophil activation involved in immune response | 34 | 5.14E-28 | 4.94E-25 |
|  | GO:0002446 | neutrophil mediated immunity | 34 | 8.07E-28 | 5.82E-25 |
|  | GO:0042119 | neutrophil activation | 34 | 1.01E-27 | 5.83E-25 |
| CC | GO:0030141 | secretory granule | 38 | 9.19E-27 | \| 2.73E-24 \| \| --- \| |
|  | GO:0042581 | specific granule | 23 | 9.77E-26 | 1.45E-23 |
|  | GO:0099503 | secretory vesicle | 38 | 2.68E-24 | 2.65E-22 |
|  | GO:0070820 | tertiary granule | 20 | 2.32E-21 | 1.72E-19 |
|  | GO:0044433 | cytoplasmic vesicle part | 42 | 5.16E-21 | 3.07E-19 |
| MF | GO:0042803 | protein homodimerization activity | 14 | 8.57E-5 | 1.94E-2 |
|  | GO:0019200 | carbohydrate kinase activity | 3 | 1.3E-4 | 1.94E-2 |
|  | GO:0003873 | 6-phosphofructo-2-kinase activity | 2 | 1.33E-4 | 1.94E-2 |
|  | GO:0003824 | catalytic activity | 38 | 1.80E-4 | 1.94E-2 |
|  | GO:0004331 | fructose-2,6-bisphosphate 2-phosphatase activity | 2 | 2.22E-4 | 1.94E-2 |
| Downregulated |  |  |  |  |  |
| BP | GO:0030098 | lymphocyte differentiation | 17 | 3.57E-16 | 7.21E-13 |
|  | GO:0030217 | T cell differentiation | 15 | 6.31E-16 | 7.21E-13 |
|  | GO:0046649 | lymphocyte activation | 22 | 3.75E-15 | 2.86E-12 |
|  | GO:0042110 | T cell activation | 18 | 9.90E-15 | 5.66E-12 |
|  | GO:0002682 | regulation of immune system process | 30 | 1.28E-14 | 5.87E-12 |
| CC | GO:0004672 | protein kinase activity | 10 | 2.13E-05 | 6.20E-3 |
|  | GO:0016773 | phosphotransferase activity, alcohol group as acceptor | 10 | 7.71E-05 | 1.12E-2 |
|  | GO:0097367 | carbohydrate derivative binding | 18 | 1.42E-4 | 1.13E-2 |
|  | GO:0004896 | cytokine receptor activity | 4 | 2.01E-4 | 1.13E-2 |
|  | GO:0038023 | signaling receptor activity | 15 | 2.10E-4 | 1.13E-2 |
| MF | GO:0098552 | side of membrane | 14 | 2.55E-08 | 5.54E-06 |
|  | GO:0009986 | cell surface | 14 | 1.55E-06 | 1.20E-4 |
|  | GO:0009897 | external side of plasma membrane | 10 | 1.66E-06 | 1.20E-4 |
|  | GO:0042105 | alpha-beta T cell receptor complex | 2 | 7.80E-05 | 3.38E-3 |
|  | GO:0044194 | cytolytic granule | 2 | 7.80E-05 | 3.38E-3 |

**Supplementary Table S4**：

Results of the KEGG enrichment analysis of DEGs (Top 5)

| Pathway ID | Pathway | Count | P-value | Genes |
| --- | --- | --- | --- | --- |
| Upregulated |  |  |  |  |
| ko05134 | Legionellosis | 5 | 9.01E-05 | NLRC4 SAR1B TLR5 CR1 NAIP |
| ko05202 | Transcriptional misregulation in cancers | 6 | 1.02E-03 | MMP9 IL1R2 GADD45A BCL2A1 HPGD ELANE |
| ko00051 | Fructose and mannose metabolism | 3 | 1.06E-03 | PFKFB2 HK3 PFKFB3 |
| ko05144 | Malaria | 3 | 4.27E-03 | HGF HBS1 CR1 |
| ko00910 | Nitrogen metabolism | 2 | 4.52E-03 | CA1 CA4 |
| Downregulated |  |  |  |  |
| ko04658 | Th1 and Th2 cell differentiation | 8 | 1.37E-07 | RUNX3 L2RB GATA3 LCK CD247 CD3E HLA-DPB1 HLA-DPA1 |
| ko04659 | Th17 cell differentiation | 8 | 3.00E-07 | RORA IL2RB GATA3 LCK CA247 CD3E HLA-DPB1 HLA-DPA1 |
| ko04640 | Hematopoietic cell lineage | 7 | 3.11E-06 | FLT3LG CD2 MS4A1 ILR7 CD3E HLA-DPB1 HLA-DPA1 |
| ko04660 | T cell receptor signaling pathway | 5 | 5.95E-05 | ITK RASGRP1 LCK CD247 CD3E |
| ko05340 | Primary immunodeficiency | 3 | 9.95E-04 | IL7R LCK CD3E |

1. **Supplementary Figure S1-S4**


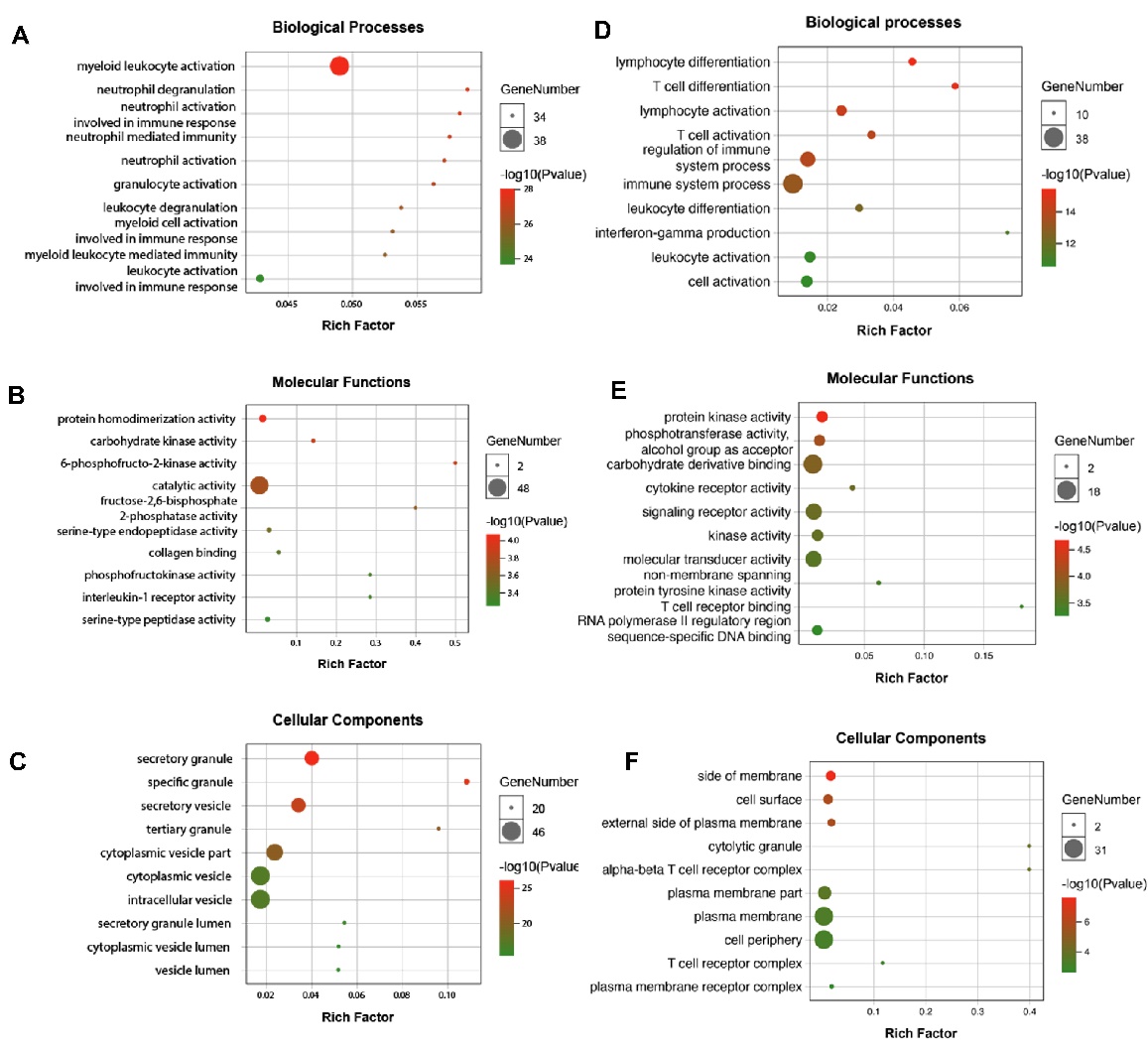


**Supplementary Figure S1:** Bioinformatic identification based on GEO database. DEGs-based pathways enriched by GO for A) biological processes, B) molecular functions, and C) cellular components. DEGs-based pathways enriched by KEGG for D) biological processes, E) molecular functions, and F) cellular components.


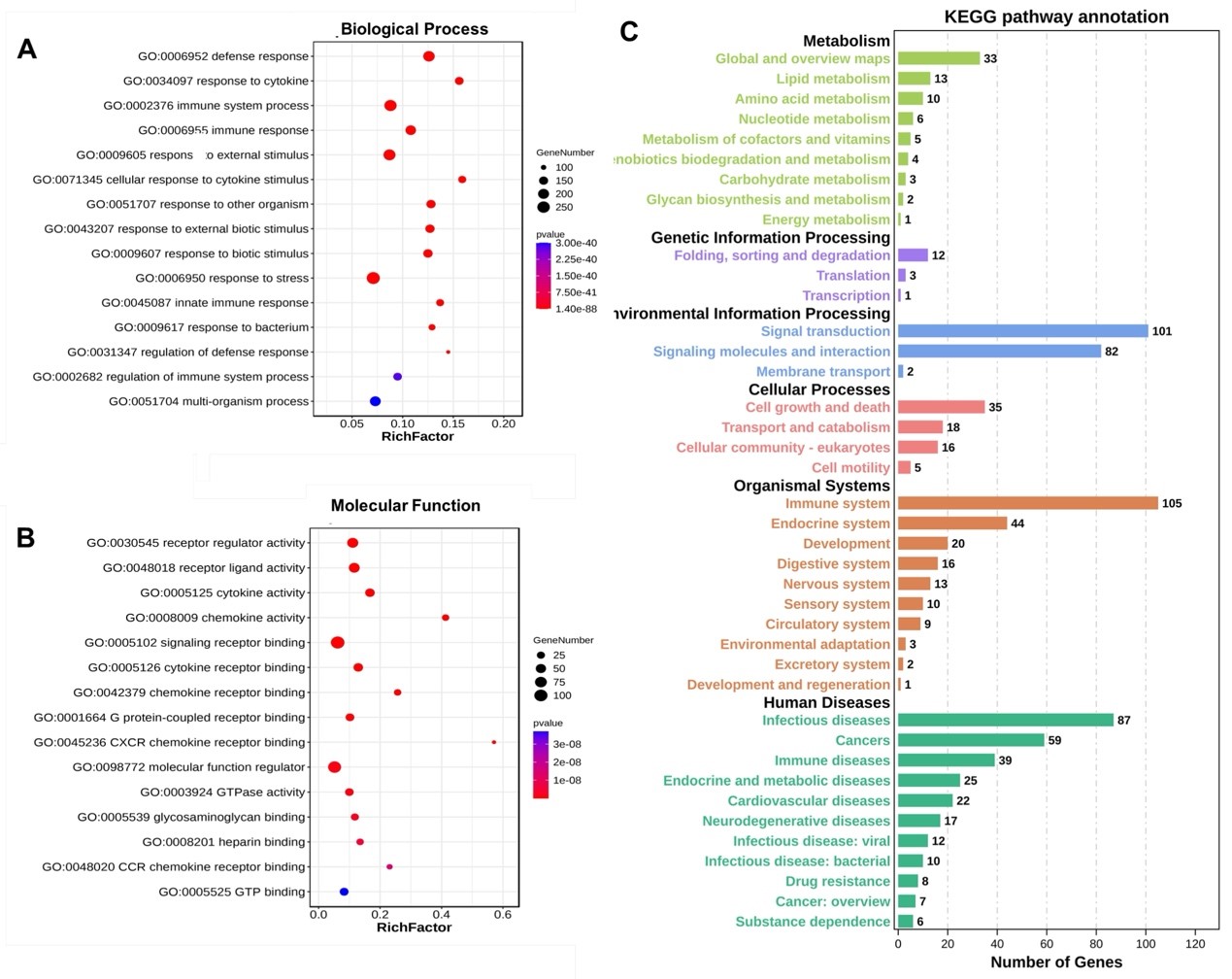


**Supplementary Figure S2:** RNA sequence bioinformatic analysis of myocardial tissue from established LPS and NC mouse model. A) DEGs-based pathways enriched by GO for biological processes; B) DEGs-based pathways enriched by GO for molecular function; C) Pathways enriched by KGEE.


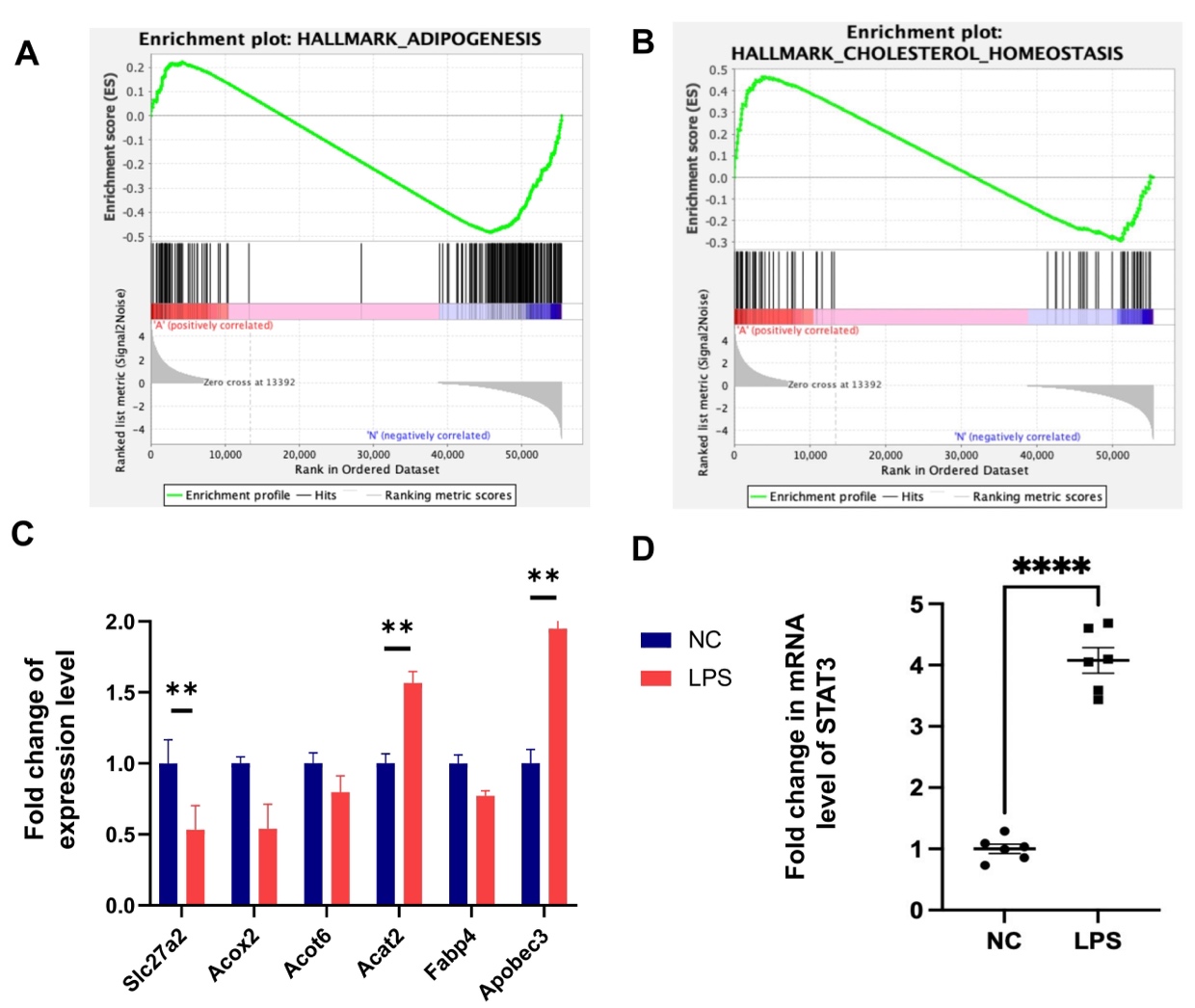


**Supplementary Figure S3:** A-B) GSEA analysis for function enrichment grouped by the high or low level of LCN-2 in the RNA-sequence analysis; C) Other molecules related to lipid metabolism changing obviously in LPS group; D) Fold change level of STAT3 in myocardial tissue.


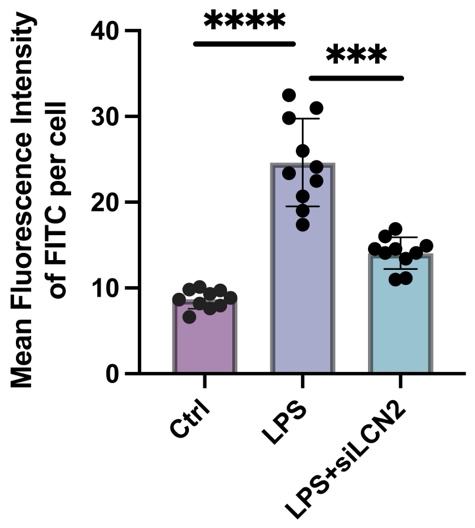


**Supplementary Figure S4:** Quantification of data of fluorescence intensity for neutral lipid.
